# Temporal features of adult neurogenesis: differences and similarities across mammalian species

**DOI:** 10.3389/fnins.2013.00135

**Published:** 2013-08-05

**Authors:** Maïna Brus, Matthieu Keller, Frédéric Lévy

**Affiliations:** ^1^INRA, UMR 85, Physiologie de la Reproduction et des ComportementsNouzilly, France; ^2^CNRS, UMR 7247Nouzilly, France; ^3^Université de ToursTours, France; ^4^Haras NationauxNouzilly, France; ^5^Laboratory of Behavioral Neuroendocrinology, GIGA Neurosciences, University of LiègeLiège, Belgium

**Keywords:** neuronal maturation, olfactory bulb, hippocampus, dentate gyrus, sub-ventricular zone, rostral migratory stream, primate, sheep

## Abstract

Production of new neurons continues throughout life in most invertebrates and vertebrates like crustaceans, fishes, reptiles, birds, and mammals including humans. Most studies have been carried out on rodent models and demonstrated that adult neurogenesis is located mainly in two structures, the dentate gyrus (DG) of the hippocampus and the sub-ventricular zone (SVZ). If adult neurogenesis is well preserved throughout evolution, yet there are however some features which differ between species. The present review proposes to target similarities and differences in the mechanism of mammalian adult neurogenesis by comparing selected species including humans. We will highlight the cellular composition and morphological organization of the SVZ in primates which differs from that of rodents and may be of functional relevance. We will particularly focus on the dynamic of neuronal maturation in rodents, primates, and humans but also in sheep which appears to be an interesting model due to its similarities with the primate brain.

## Introduction

Adult neurogenesis is conserved throughout evolution, from crustaceans (Beltz and Sandeman, 2003; Schmidt, 2007a,b), to insects (Scotto-Lomassese et al., 2003; Cayre et al., 2007) and to higher vertebrates, including fishes (for review see Kizil et al., 2011), reptiles (Font et al., 2001), birds (Goldman and Nottebohm, 1983), and mammals with species ranging from rabbit (Fasolo et al., 2002; Luzzati et al., 2006), guinea-pig (Guidi et al., 2005), mice (Hinds, 1968; Lois and Alvarez-Buylla, 1994), rats (Altman and Das, 1965a,b), cats (Das and Altman, 1971), dogs (Blakemore and Jolly, 1972; Hwang et al., 2007), cow (Rodriguez-Perez et al., 2003), sheep (Hawken et al., 2009; Brus et al., 2010, 2013; Migaud et al., 2010), non-human primates (Gould et al., 1999; Kornack and Rakic, 1999; 2001) to humans (Eriksson et al., 1998; Curtis et al., 2011). Most studies have been carried out on rodent models and demonstrated that a production of new neurons continues throughout life mainly in two main structures in most mammals, namely the dentate gyrus (DG) of the hippocampus and the main olfactory bulb (MOB) (for review see Ming and Song, 2005). In the DG, progenitor cells proliferate in the sub-granular zone (SGZ) and give rise to neuroblasts that migrate in the granule cell layer (GCL) to become new mature neurons and integrate the neuronal network of the hippocampus (Nogues et al., 2011). In the MOB, newly-born cells originate from the sub-ventricular zone (SVZ) where neural stem cells give rise to transient amplifying cells which differentiate into neuroblasts. These neuroblasts migrate along the rostral migratory stream (RMS) to reach the MOB where they become local inhibitory interneurons and integrate neural networks of the olfactory system (Lledo and Saghatelyan, 2005).

At present, species with high reproductive rates, rapid development, and short life span (e.g., rats and mice) have been closely studied to gain insights into the functioning of adult neurogenesis in a therapeutic perspective on neurodegenerative diseases or on cell replacement therapy in humans. By contrast, species with long development and life span have not been well-studied (e.g., marmots, elephants, cows, sheep, and long-lived primates such as humans), and if adult neurogenesis is well preserved throughout evolution, some of its features differ between species. Indeed, timing of generation, migration, and differentiation of new neurons appear to differ according to brain size and lifespan (Cameron et al., 1993; Kornack and Rakic, 2001; Petreanu and Alvarez-Buylla, 2002; Winner et al., 2002; Brown et al., 2003; Steiner et al., 2004; McDonald and Wojtowicz, 2005; Snyder et al., 2009; Kohler et al., 2011; Sawamoto et al., 2011; Brus et al., 2013).

The present review therefore proposes to target the differences in the mechanism of adult neurogenesis by comparing some mammalian species including humans. We will particularly focus on the dynamic of neuronal maturation in rodents, primates and humans but also in sheep which appears to be an interesting model due to its similarities with the primate brain.

## SVZ organization and migration pathway (Figure 1)

Adult neural stem cells reside in the SVZ and give rise to neurogenic and gliogenic precursors (Doetsch et al., 1999) which then migrate toward the MOB forming a pathway called the RMS. The RMS is composed of migrating neuroblasts and astrocytes which express various molecular factors favoring cell migration (Lois et al., 1996; Gritti et al., 2002). When reaching the MOB, newly-born cells migrate radially, differentiate into interneurons and integrate mainly the granular cell layer (95%) but also the glomerular layer (5%) (Lledo et al., 2006). Studies report differences between rodents and primates in the distribution of cell population and the cytoarchitecture of the SVZ that may be of functional relevance (Gil-Perotin et al., 2009). In the adult marmoset and the cynomulgus monkey (*Macaca fascicularis*), the SVZ have a three layer organization, with the ependymal layer surrounding the lateral ventricles, a hypocellular gap layer formed by astrocytic and ependymal expensions, and an astrocyte ribbon layer composed of astrocytic bodies. However, the hypocellular layer is very thin or absent in the adult anterior SVZ (Gil-Perotin et al., 2009; Sawamoto et al., 2011). Similarly to non-human primates, tree layers are observed in humans throughout the lateral ventricular wall with varying thickness and cell densities: a monolayer of ependymal cells, a hypocellular gap layer, a ribbon of cells composed of astrocytes, and a transitional zone into the brain parenchyma (Quinones-Hinojosa et al., 2006). The ribbon of SVZ astrocytes lining the lateral ventricles proliferates *in vivo* and behaves as multipotent progenitor cells *in vitro* (Sanai et al., 2004). By contrast in adult mice, the presence of a hypocellular gap layer is not established and the SVZ is composed of neuroblasts, glial cells, and putative precursor cells. Migrating neuroblasts were isolated from the ependymal cells and striatum by two ultrastructurally distinct astrocytes which ensheated new neuroblasts. Transient amplifying cells which divide themselves actively formed focal clusters closely associated with chains of neuroblasts (Doetsch et al., 1997). In bovines, the morphological arrangement of the various cell types is different from that described in rodents and partly resembles results reported in humans. The lateral ventricle wall is composed of three layers the ependymal, subependymal, and subjacent glial layers which are more or less present along the rostro-caudal axis (Rodriguez-Perez et al., 2003). As in humans, the SVZ glial network appears reduced to an incomplete ribbon separated from the ependymal by a hypocellular gap layer (Rodriguez-Perez et al., 2003; Bonfanti and Peretto, 2011). In sheep and rabbit, the SVZ is particularly expanded to the open olfactory ventricle in the MOB (Luzzati et al., 2003; Brus et al., 2013), suggesting that the migration pathway of adult-born cells follows this ventricle. In sheep, the presence of a hypocellular-like layer which separates chains of neuroblasts from the ependymal layer is reported only in the anterior part of the SVZ, suggesting some similarity with bovines and primates (Figure 2A; Brus et al., 2013).

**Figure 1:**
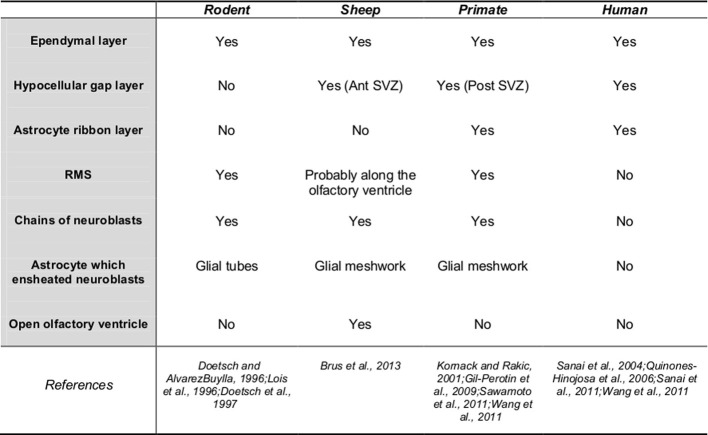
**Sub-ventricular zone organization and migration pathway to the main olfactory bulb.** Ant SVZ, anterior sub-ventricular zone; post-SVZ, posterior sub-ventricular zone; yes, presence; no, absence.

**Figure 2:**
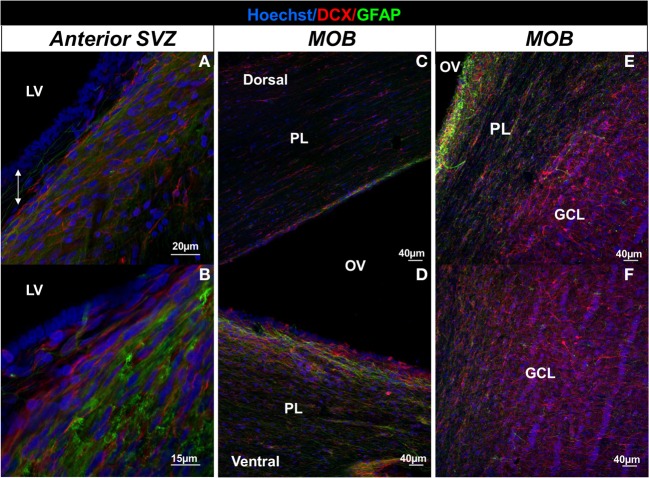
**Illustration of the migration pathway from the SVZ to the MOB in the sheep brain.** Confocal images depicting fluorescent immunolabeling of DCX (red) and GFAP (green) in the anterior SVZ **(A,B)**, in the dorsal part **(C)** and the ventral part **(D)** of the MOB, and in the granular cell layer of the MOB in sheep **(E,F)**. Nuclei of cells are revealed by a Hoechst staining (blue). Note the presence of a hypocellular-like layer between the ependymal layer lining the lateral ventricle and chains of neuroblasts in the anterior SVZ (double white arrow in **A**) and the shift of DCX+ cells that are oriented parallel to the olfactory ventricle in the periventricular layer **(E)** and perpendicular to rows of granular cell nuclei in the GCL of the MOB **(E,F).** GCL, granular cell layer; LV, lateral ventricle; PL, periventricular layer; OV, olfactory ventricle.

In rodents and non-human primates, the majority of migrating cells coming from the SVZ migrates along each other forming chains tangentially oriented to the ventricle. These chains of neuroblasts are ensheated by astrocytes which formed glial tubes in rodents and glial meshwork in monkey (Doetsch and Alvarezbuylla, 1996; Lois et al., 1996; Sawamoto et al., 2011; Wang et al., 2011). Like non-human primate, sheep seems to possess chains of neuroblasts in the anterior SVZ which are immersed within an astrocytic meshwork oriented in parallel direction to the longitudinal axis of the lateral ventricle (Figures 2A,B) and are more concentrated in the ventral portion of the SVZ as observed in primates (Gil-Perotin et al., 2009; Sawamoto et al., 2011; Wang et al., 2011; Brus et al., 2013). In sheep, injection of an adenovirus coupled with a promoter that induces the synthesis of a fluorescent protein (eGFP) has revealed a migration pathway from the SVZ up to the MOB which followed the lateral ventricle from the SVZ to the ventricle of the olfactory bulb (Brus et al., 2013). As in rodents and non-human primates, chains of neuroblasts enter the MOB predominantly by its ventral part (Figures 2C,D) and seem to migrate radially to the granular cell layer, as neuroblasts with an elongated morphology are located perpendicular to the granular cells (Figures 2E,F) (Brus et al., 2013).

In humans, very few new neuroblasts appear to be produced in the SVZ (Sanai et al., 2011). The presence of a RMS and newborn neurons in the adult human SVZ seems non-existent (Sanai et al., 2004, 2007, 2011; Quinones-Hinojosa et al., 2006; Wang et al., 2011). Only the anterior part of the SVZ contain some elongated Tuj1+ cells (a marker of migrating neuroblasts) with a migratory morphology but no evidence of chains of migrating neuroblasts in the SVZ or in the pathway of the olfactory bulb was found (Sanai et al., 2004; Quinones-Hinojosa et al., 2006). Some cells in the rostral subependymal zone of human brain expressed the polysialic acid form of neural cell adhesion (PSA-NCAM), characteristic of migrating neuroblasts, suggesting that some neuroblasts-like cells exist in the human subependymal zone (Weickert et al., 2000). Interestingly, no neuroblast is specifically found in the human olfactory bulb (Wang et al., 2011). However, the infant human SVZ and RMS contain an extensive corridor of migrating immature neurons before 18 months of age but this neurogenesis is nearly extinct by adulthood (Sanai et al., 2011). Only one research group reports the presence of a human RMS composed by migrating neuroblasts which is organized around a lateral ventricular extension reaching the olfactory bulb called the ventriculo-olfactory neurogenic system (VONS) (Curtis et al., 2007; Kam et al., 2009). However, others authors report a continuous structure extending from the SVZ to the olfactory bulb within the adult human brain but demonstrate that this olfactory ventricle collapses during fetal development (Sanai et al., 2007, 2011). Moreover, it appears that the VONS was previously described and called “ventral lateral extension” (Bernier et al., 2000; Weickert et al., 2000) and characterized as the “SVZ-olfactory trigone connection” (Sanai et al., 2004) which is a region where the olfactory ventricle existed early in human development.

## The main olfactory bulb and the rostral migratory stream as neurogenic zones?

The distinction between neurogenic and non-neurogenic region is based on a small number of fundamental studies using transplantation approaches, production of neurosphere cultures from animal tissue and identification of neural stem cells by *in situ* immunolabeling. For instance, implantation of hippocampal precursor cells in the RMS of rat induced a production of olfactory interneurons indicating that the migration pathway has a neurogenic potential (Suhonen et al., 1996). Moreover, during migration new neuroblasts in rats continue to divide themselves and initiate neuronal maturation. Proliferation, however, is much slower than in the SVZ and the cell cycle duration is lengthened. Interestingly, unlike the progenitors that divide within the SVZ and generate more granule cells than periglomerular cells, the proliferating cells within the migratory pathway generate more periglomerular cells than granule cells (Smith and Luskin, 1998).

The presence of neural precursors within the MOB has been also reported in mice (Gritti et al., 2002), sheep (Brus et al., 2010, 2013), macaque monkey (Kornack and Rakic, 2001) and humans (Pagano et al., 2000; Bedard and Parent, 2004), suggesting a contribution to the addition of newborn neurons to the olfactory system. In mice multipotential precursors with stem cell features can be isolated from the entire rostral extension of the SVZ but also from the distal portion within the olfactory bulb (Gritti et al., 2002). In the macaque monkey, 2 h after BrdU injections, labeled cells are observed in the MOB in the white matter of the core and interneuron layers and BrdU-labeled figures are occasionally detected (Kornack and Rakic, 2001). In human, stem cells have been isolated from the olfactory bulb of adults. These cells could proliferate *in vitro*, retaining all of the typical characteristics of neural stem cells; they also have the ability to give rise to new neurons, astrocytes and oligodendrocytes (Pagano et al., 2000). A more recent study shows that cells labeled with proliferative markers such as Ki67 and PCNA (proliferating-cell nuclear antigen) co-expressed markers of immature neuronal state such as DCX and NeuroD but also a marker of neural stem cells Nestin, suggesting that in human, the MOB could also produce adult newborn cells (Bedard and Parent, 2004). Likewise in the MOB of sheep, the presence of BrdU-positive cells co-labeled with GFAP or Sox2, another markers of neural stem cells, further suggesting that the production of new olfactory neurons can also occur locally (Brus et al., 2010, 2013). Finally, in mice an *in vitro* study shows that stem cells isolated from the olfactory bulb generated about 10 times less neurospheres than those from the SVZ (Gritti et al., 2002). Thus although the MOB could constitute a source of progenitors in different species, its contribution to the production of new neurons appears to remain marginal.

## Dynamics of maturation of the newborn neurons

**Figure 3:**
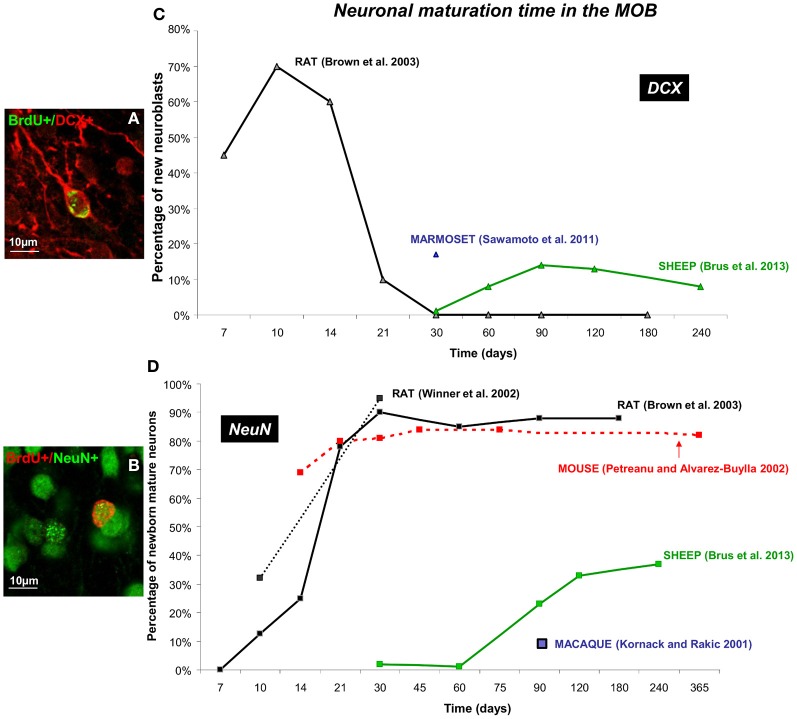
**Dynamic of neuronal maturation time of adult-born cells in the MOB. (A,B)** Confocal images of double fluorescent immunolabeling of BrdU (green)/DCX (red), a marker of migrating neuroblasts **(A)** and BrdU (red)/NeuN (green) a marker of post-mitotic neurons **(B)** in the granular layer of the MOB in sheep. **(C,D)** Neuronal maturation time of different mammalian species (mouse, rat, sheep, marmoset, and macaque) are represented as percentage of newborn neuroblasts **(C)** and newborn neurons **(D)** across different survival times (Kornack and Rakic, 2001; Petreanu and Alvarez-Buylla, 2002; Winner et al., 2002; Brown et al., 2003; Sawamoto et al., 2011; Brus et al., 2013). Note that maximal neuronal maturation is reached in the first month post-injection in mice and rat whereas in sheep and primate the first new mature neurons are only observed at 3 months (90 days). The blue square represent the first adult-born neurons (BrdU+/NeuN+) observed in the MOB of the macaque monkey but no percentage was specified (Kornack and Rakic, 2001).

**Figure 4:**
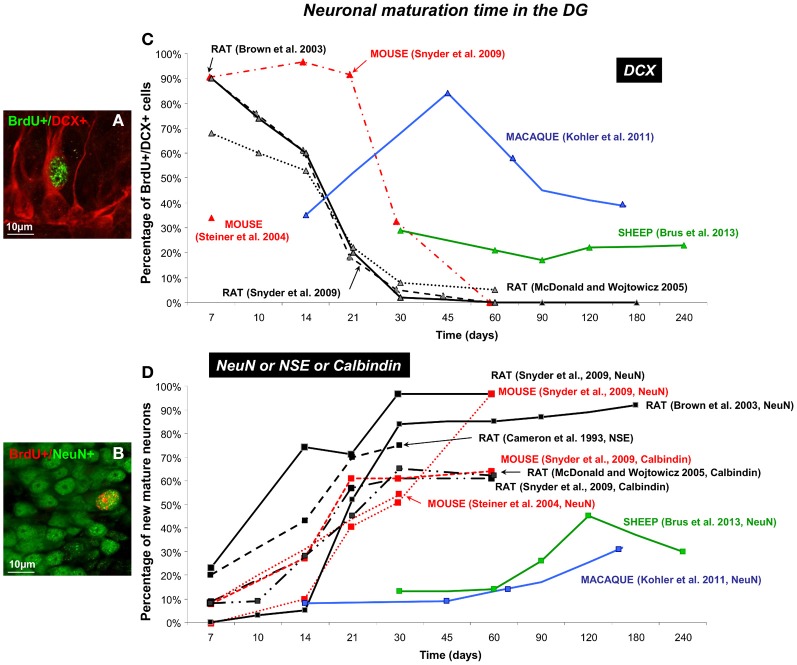
**Dynamic of neuronal maturation time of adult-born cells in the DG. (A,B)** Confocal images of double fluorescent immunolabeling of BrdU (green)/DCX (red), a marker of migrating neuroblasts **(A)** and BrdU (red)/NeuN (green) a marker of post-mitotic neurons **(B)** in the granular layer of the DG in sheep. **(C,D)** Neuronal maturation time of different mammalian species (mouse, rat, sheep and macaque) are represented as percentage of newborn neuroblasts **(C)** and newborn neurons **(D)** across 240 survival days reported by several studies (Cameron et al., 1993; Brown et al., 2003; Steiner et al., 2004; McDonald and Wojtowicz, 2005; Snyder et al., 2009; Kohler et al., 2011; Brus et al., 2013). Note that maximal neuronal maturation is reached in the first month post-injection in mice and rat whereas in sheep and primate new immature and mature neurons appear around 15–30 days post-injection and reach a maximum at 4 months in sheep and around 6 months in macaque monkey.

**Figure 5:**
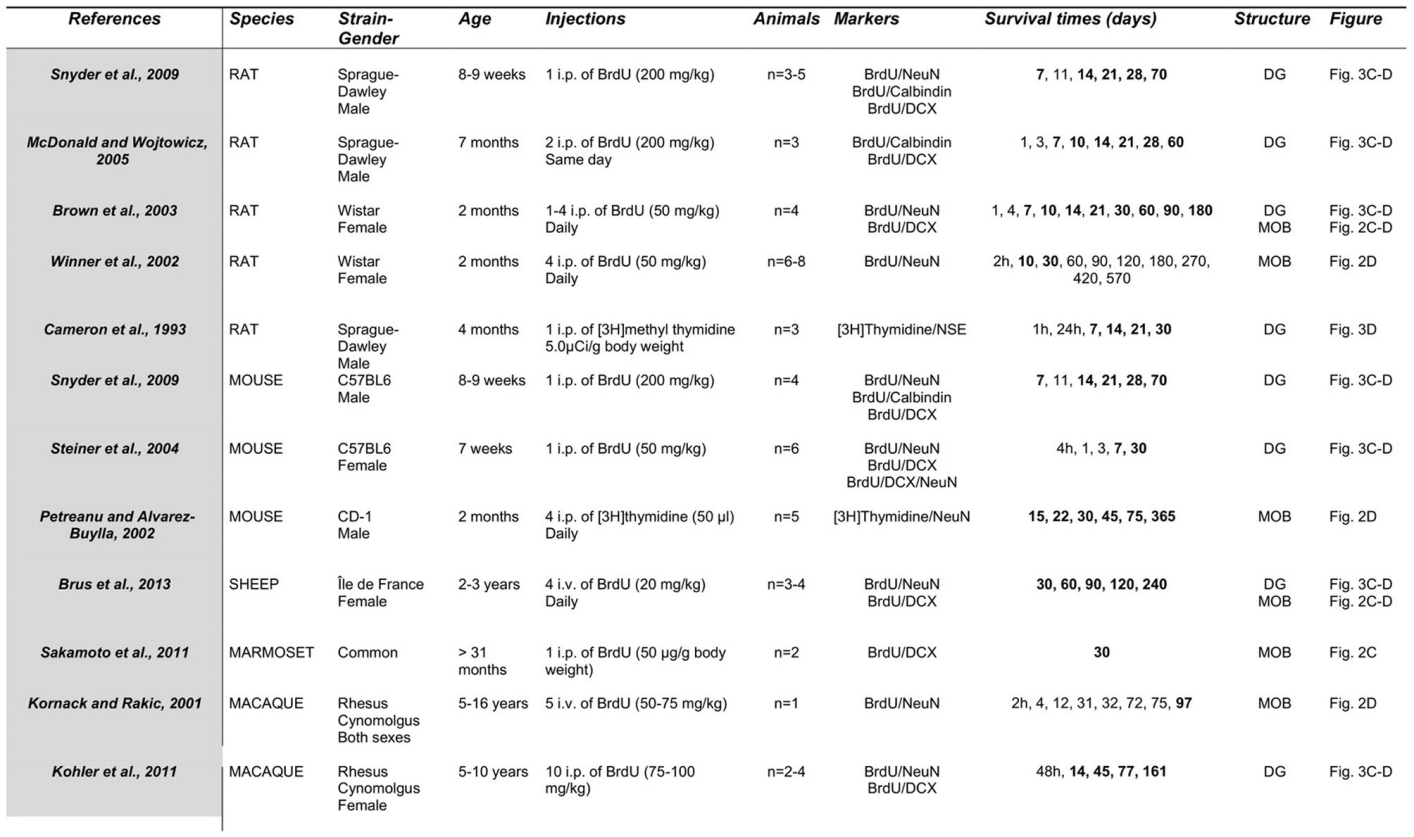
**Comparative study of protocols used to estimate neuronal maturation of adult born neurons.** Times in bold correspond to those represented in Figures 3, 4. Number of animals corresponds to the sample size by groups. BrdU, bromodeoxyuridine; DCX, Doublecortin; NeuN, neuron-specific nuclear protein; NSE, neuron specific enolase; DG, dentate gyrus; MOB, main olfactory bulb.

Over the last 10 years, detection of newborn cells has been generally based on the incorporation of the exogenous proliferation marker Bromodeoxyuridine (BrdU) into the DNA of dividing precursor cells. Animals are sacrificed at different times of cell survival and immunohistochemical techniques are used to visualize the BrdU. This immunolabeling is associated with the most commonly used marker of mature and post-mitotic neurons NeuN (neuron-specific nuclear protein) (Figures 3B, 4B), NSE (neuron specific enolase) or Calbindin (a calcium binding protein), and doublecortin (DCX) or β 3-tubulin (TuJ1) for migrating neuroblasts (Figures 3A, 4A). Using these various markers, differences in the time course of neuronal maturation in the MOB and in the DG have been reported between rodents, primates and sheep (Figures 3, 4). The disparity between the points of investigation across species is remarkable. Indeed, the rodent studies of adult neurogenesis largely center upon very young animals (around 2 months of age) whereas primate studies focus more on young adult to middle age (5–16 years; Figure 5). Dynamics of maturation of newborn cells could differ according to age and this might alter interpretations, especially when rodent studies aimed at translational research for human cellular therapy.

### The main olfactory bulb

In the rat brain, 10 days after BrdU labeling approximately 70% of the BrdU-positive cells express the marker of migrating neurons DCX (Brown et al., 2003) whereas the first co-localization with the NeuN marker is just detected in the granular layer of the MOB (Winner et al., 2002; Brown et al., 2003) (Figures 3C,D). The population of new mature neurons increases to nearly 90% by 30 days post-injection, while number of cells expressing DCX decreases to very low levels. At the last time point analyzed (180 days) the proportion of differentiated neurons remains high, around 90% whereas BrdU+/DCX+ cells are undetectable (Brown et al., 2003). From 90 days old up to 19 months old, 50% of the newly generated granule cells survive (Winner et al., 2002). Similarly in mice, the maximum number of labeled mature neurons (>80%) is observed around 30 days after injection of [3H] thymidine, another marker of proliferating cells (Petreanu and Alvarez-Buylla, 2002). However, from 15 days after BrdU injection, the proportion of newborn neurons appears to be already high (69%) contrasting with rat (25%) (Brown et al., 2003). Between days 15 and 45 after birth, soon after the development of spines, the number of [3H] thymidine labeled cells declined by 50%. However, the cells which survive the first 2 months persist for up to 1 year. The proportion of newborn neurons remains stable until the last survival point at 365 days demonstrating that newborn cells could survive for a long time in the rodent MOB (Petreanu and Alvarez-Buylla, 2002).

By contrast to rodents, in sheep the highest proportion of new mature neurons is found 240 days (8 months) after BrdU injections (37%) and no mature neurons is observed before 90 days post-injections (3 months) (Figure 3C) (Brus et al., 2013). Very few neuroblasts are found at 30 days after BrdU injections (14%) and this population reaches a maximum at 90 days post-injections and remains stable until the last survival time (240 days) (Brus et al., 2013). Similarly in primates, only 17% of newborn cells express the DCX marker at 30 days post-injection in the marmoset brain (Sawamoto et al., 2011) (Figure 3C). In the macaque monkey, none of the BrdU positive cells in the MOB express the mature neuronal marker NeuN at 32 and 75 days after BrdU injections. However, at 75 days post-injection, a large population of BrdU+/Tuj1+ cells is present in the RMS as it entered the MOB, suggesting that new cells begin to differentiate. This apparent surge of new cells is not observed at an earlier survival time (32 days). By 97 days post-injection, some of the BrdU positive cells in the macaque granular layer are immunoreactive for NeuN and are similar in size and shape to adjacent NeuN positive granule neurons. These results indicate the presence of newly generated neurons in the MOB of sheep and primates, Contrary to rodents, these cells do not appear to be differentiated until 90 days of age indicating that in these species neuronal maturation is a much longer process than in rodents (Kornack and Rakic, 2001; Brus et al., 2013).

### The dentate gyrus of the hippocampus

In the SGZ of the common marmoset, robust staining is observed for protein labeling transient stages of neuronal differentiation (GFAP, Sox2, DCX, Tuj1, NeuN) (Bunk et al., 2011). Their expression pattern is similar to the one observed in rodents suggesting that expression profiles during neuronal differentiation are conserved between these species during adulthood. However, the dynamic of neuronal maturation of the newborn cells appears to be different. Indeed, in the DG of the rat, the highest percentage of BrdU positive cells expressing DCX (70–90%) is reached at 7 days post-injection, thereafter DCX expression is rapidly down-regulated during the next 2 weeks (Figure 4C). DCX expression was observed in only 2–7% of BrdU-positive cells by 30 days and became undetectable by 60 days after labeling (Brown et al., 2003; McDonald and Wojtowicz, 2005; Snyder et al., 2009). As expected and in parallel with the decline of DCX expression, BrdU-labeled cells immunoreactive for NeuN are first detected in the hippocampus at 7 days post-injection and this proportion increases to approximately 80–95% 30 days after labeling. This percentage stays stable around 95% for the last time point analyzed at 180 days (Brown et al., 2003; Snyder et al., 2009) (Figure 4D).

By using another markers of mature neuron (Calbindin, NSE), slightly different percentages are observed but the temporal pattern of neuronal maturation appears similar (Figure 4D). In rats, BrdU+/Calbindin+ cell counts increase steadily between 10 and 30 days after injection, remain steady over 70 days; however, the maximal proportion of new mature neurons does not exceed 65% in both rats and mice (Cameron et al., 1993; McDonald and Wojtowicz, 2005; Snyder et al., 2009). Even at the longest time points examined, approximately 40% of BrdU+ cells do not express calbindin. Mice and rats showed no differences in the expression of calbindin at any time point (McDonald and Wojtowicz, 2005; Snyder et al., 2009).

Finally, at 7 days following injection, 20% of 3H thymidine labeled cells are NSE-immunoreactive in the DG of rats (Cameron et al., 1993). This proportion increases rapidly to reach a maximum at 30 days where the majority of H3 thymidine labeled cells are NSE-immunoreactive (75%) and are virtually indistinguishable from neighboring granule neurons (Cameron et al., 1993).

In mice, dynamic of expressions of DCX marker by BrdU positive cells differs between studies. Like in male rats where a high proportion of BrdU positive cells expresses the marker of migrating neuroblasts at 7 days post-injection (90%), male C57BL6 mice show 89% of co-labeling (Snyder et al., 2009) whereas in female C57BL6 mice only 19% of all BrdU positive cells expressed DCX and 14% co-expressed DCX and NeuN (Steiner et al., 2004) (Figures 4C, 5). These results suggest a difference between genders in neuronal maturation time in the DG. In both rats and mice, the percentage of DCX expressing cells dropped to zero, but the time course of this decrease was different between species (Brown et al., 2003; McDonald and Wojtowicz, 2005; Snyder et al., 2009). More BrdU positive cells were DCX positive in mice than in rats at 7, 14, 21, and 30 days, a difference that was especially pronounced at 21 days, when 90% of BrdU positive cells express DCX in mice, compared with only 20% of cells in rats (Brown et al., 2003; McDonald and Wojtowicz, 2005; Snyder et al., 2009). Similarly to rats, the proportion of new cells which expresses the NeuN marker at 7 days in mice is low (0–8%) (Figure 4D) and increases until 30 days where most of the BrdU labeled cells have differentiated into advanced stages of cellular development (Steiner et al., 2004; Snyder et al., 2009). Indeed, in the neuronal population, around 50% of the BrdU positive cells express NeuN but this proportion remains lower than that observed in rats (90%) (Steiner et al., 2004; Snyder et al., 2009) (Figure 4D). These results show some differences in the amount of cells which differentiate into neurons between mice and rats, however the dynamic of neuronal maturation remains similar and occurs within the first month after birth. Interestingly, using a low criterion of fluorescence intensity of NeuN staining, Snyder et al. found that nearly all 1-week old BrdU positive cells expressed NeuN in both mice and rats (Snyder et al., 2009). This suggests that one must be careful when comparing studies since the percentage of double staining appears to be dependent on methodological conditions.

Neuronal maturation in the DG appears to be longer in sheep than in rodents, similarly to what was observed in the MOB. The highest proportion of new mature neurons (45%) is found at 4 months after BrdU injections, even if a small proportion of BrdU+ cells (13%) already shows a mature neuronal phenotype at 1 month post-injection (Brus et al., 2013) (Figure 4D). Contrary to rodents, the highest proportion of neuroblasts (29%) appears only at 30 days after BrdU injections and is stable up to 240 days survival time (Brus et al., 2013). Similarly in the macaque monkey, the maximal proportion of BrdU+/NeuN+ cells is observed around 160 days and less than 10% of new neurons can be observed at 14 days post-injection (Kohler et al., 2011). At the same time, 35% of BrdU+ cells express the DCX marker of immature neurons. This proportion increases to reach a maximum at 45 days (84%) whereas in rodents the maximum is reached at 7 days post-injection (Brown et al., 2003; Snyder et al., 2009) (Figure 4C). Then, this proportion decreased to 38% at 160 days post-injection suggesting that the process of maturation is not yet complete (Kohler et al., 2011).

Taken together, these results reveal fundamental differences in the time course of neuronal maturation between rodents, sheep and primates and in particular a significantly longer time of differentiation in sheep and in non-human primates. However, the highest proportion of new mature neurons observed in sheep and primates is lower than 50% of the total of BrdU+ cells indicating that a majority of new neurons had not yet fully matured. The presence of a large pool of undifferentiated newborn cells which expressed Sox2, a marker of neural stem cells and progenitor cells up to 8 months of survival time in the DG of sheep support this hypothesis (Brus et al., 2013).

In humans, there is only one single study looking at neurogenesis in the DG with BrdU (Eriksson et al., 1998) and very few data on the dynamic of neuronal maturation are available. Ethical issues limit administration of BrdU to humans and experiments with different timing of cell survival are impossible. Moreover, BrdU labeling in fluorescent studies is hampered by the autofluorescent staining of lipofuscin that is especially high in the brain of older subjects in which most studies are performed. Finally, while adult neurogenesis has been shown to occur across the lifespan in the DG (Eriksson et al., 1998), neurogenesis declined exponentially with increasing age (Knoth et al., 2010) rendering the study of neural maturation more difficult in humans for which collection of brain tissues is limited. However, one study conducted on patients with a mean age of 64 ± 3 years reports 22% of BrdU/NeuN and 23% BrdU/NSE double labeled cells in the DG after 16–781 days (~2 years) post-injections delays (Eriksson et al., 1998). These data indicate that timing of maturation of new neurons would be longer in human than in rodents highlighting the importance of studying the features of adult neurogenesis in other models than rodents, like sheep and primates, especially when aimed at translational research for human cellular therapy.

## Conclusion

Cross-species studies on adult neurogenesis reviewed in the present manuscript reveal some differences especially concerning the cytoarchitecture of the SVZ and the dynamic of neuronal maturation. The distribution of cell population in the SVZ appears to differ between rodents and primates. In human and non-human primates, the SVZ have a three layer organization with a hypocellular gap layer and an astrocytic ribbon whereas in rodents, no gap layer is observed and migrating neuroblasts are ensheathed by a glial-tube formed by astrocytes. Sheep present similarities with the primate brain, with the presence of a hypocellular-like layer in the anterior SVZ and chains of neuroblasts which are immersed within an astrocytic meshwork. Some similarities are also observed between species as different studies in rodents, sheep, primates and humans report evidence for a neurogenic constitution of the RMS and the OB, which contribute to the production of new neurons in the olfactory system.

Finally, the maturation time of new neurons appears to be much longer in primates and sheep than in rodents in the MOB and the DG. In humans, because no complete series through the entire DG could be obtained, no absolute counts of BrdU-labeled neurons could be generated (Eriksson et al., 1998). Indeed, estimate precisely the production of new neurons in the adult human brain is difficult. The complexity in the cortex development, lisencephalic (e.g., mice and rat) or gyrencephalic brains (e.g., sheep, macaque, humans) and life expectancies could be involved in the differences observed. It is critical to also study species that span both the phylogenetic spectrum and the gamut of longevity from short-lived to long-lived species. At present, no data are available on the neuronal maturation duration in humans and further investigations would be necessary to know whether the timing of differentiation of new neurons is as long in humans as in non-human primates or sheep. However, several studies report little or extinct neurogenesis, at least in the olfactory system, in human adulthood raising the potential limitation to the attempt of using endogenous neural stem cells in replacement therapies following injury to the human brain.

### Conflict of interest statement

The authors declare that the research was conducted in the absence of any commercial or financial relationships that could be construed as a potential conflict of interest.
